# Intervention to Reduce Anxiety Pre- and Post-Eye Surgery in Pediatric Patients in South Korea: A Preliminary Quasi-Experimental Study

**DOI:** 10.3390/children9010065

**Published:** 2022-01-04

**Authors:** Hyeran Yi, Hanna Lee

**Affiliations:** 1 Department of Nursing, Pusan National University Yangsan Hospital, Yangsan-Si 50612, Korea; yhr1113@gmail.com; 2 Department of Nursing, Gangneung-Wonju National University, Wonju-Si 26403, Korea

**Keywords:** anxiety, child, education, information, surgery

## Abstract

In this study, we aimed to identify the effect of preoperative information on postoperative anxiety among children undergoing one-day eye surgery. We utilized a nonequivalent control group and a pretest–posttest quasi-experimental design. The participants were 15 children in the experimental group and 15 children in the control group. Preoperative information was provided to the experimental group in the waiting room. Anxiety level was measured using the Children’s Emotional Manifestation Scale and pulse rate. For pulse rate, there were no statistically significant differences between the groups. In the behavioral anxiety response, there were statistically significant differences between the experimental and control groups (Z = −4.15, *p* < 0.001). The results suggest that the provision of preoperative information can be an effective intervention for reducing postoperative anxiety and improving the health of children undergoing surgery.

## 1. Introduction

Childhood is a crucial period for the development of vision, and the timely treatment of all eye diseases is critical [1]. For example, strabismus—abnormal eye alignment that hinders uniform bilateral eye movement [2]—is a risk factor for amblyopia during vision development. If left untreated, amblyopia and impaired binocular vision can lead to diminished stereovision [1,2]. Similarly, to facilitate the recovery and growth of visual functions disrupted by congenital epiblepharon—a condition that causes the eyelashes to irritate the cornea and induces astigmatism—aggressive treatment, such as surgery, is important [1,3].

However, surgery is a dreadful event for children, as they may experience intense anxiety; physical injury, separation from parents, uncertainty, and the loss of autonomy all contribute to this anxiety [4,5]. Anxiety in children during surgery may elevate their pain, affect their vital signs, and hinder their rapport with health care providers, thereby delaying recovery [6]. In particular, children undergoing eye surgery not only feel uncomfortable but may also experience anxiety due to their vision being limited after the postoperative application of ophthalmic ointment and eye patch [7].

Children are capable of expressing their emotions, thoughts, and opinions, and they want to receive information about their surgery directly from an expert [8,9]. This enables them to understand in advance what to expect from a threatening event, thereby relieving their preoperative and postoperative anxiety; reducing these anxieties can facilitate recovery and promote their return to normal life [8,10,11,12].

A study that administered an education program using animations to children undergoing strabismus surgery and their caregivers [13] reported that it reduced preoperative anxiety and increased nursing satisfaction. Educational interventions with visual aids and in-person conversations have been effective in reducing anxiety in patients undergoing surgery [14], and preoperative nursing interventions utilizing smartphones [15], pictures [12], and clown-nurse interventions [16] have effectively reduced preoperative anxiety. However, for most eye surgeries, the entire process is completed within a few hours, leaving little time for pre- and postoperative care [13]. Furthermore, surgery-related information is generally delivered to parents, and information sheets for children are often lacking [12]. Most research has focused on reducing anxiety before surgery [12,13,15], but in the case of ophthalmic surgery, the post-surgery period is particularly significant. In a previous study [16] that conducted educational interventions for children undergoing strabismus surgery, the experimental group experienced a decrease in postoperative anxiety behavior scores compared to the control group. As the content of preoperative information provided to children is often based on health care providers’ experience or existing literature, it is important to identify the anxiety risk factors and educational needs of children, the actual targets of the information. Hence, we investigated the effects of preoperative information delivery on postoperative anxiety in children undergoing same-day eye surgery.

## 2. Materials and Methods

### 2.1. Study Design

**Figure 1:**
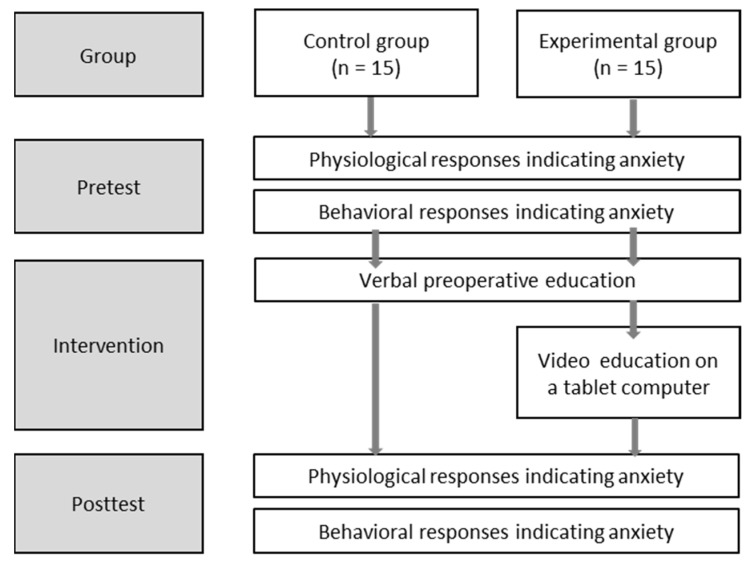
Process flow diagram.

This quasi-experimental study used a nonequivalent control group pretest–posttest design to investigate the effects of preoperative information delivery on postoperative anxiety relief in children undergoing same-day eye surgery (Figure 1).

### 2.2. Participants

The participants were children admitted to a tertiary hospital in the Republic of Korea for same-day eye surgery between 19 June and 16 October 2018. The 30 enrolled participants (15 in the control group and 15 in the experimental group), aged 5–11 years, met the following inclusion criteria: (1) informed consent provided by both child and caregiver; (2) American Society of Anesthesiologists class I (healthy without systemic disease); (3) capable of communication; (4) scheduled for strabismus surgery at the hospital; (5) capable of understanding information provided before surgery. The exclusion criteria were (1) any brain injury or visual, hearing, or emotional impairment and (2) severe illness or an emergency condition.

### 2.3. Instruments

#### 2.3.1. Educational Intervention

The structure of the educational intervention was based on children’s anxiety risk factors and their educational needs, determined from the 23–30 May 2018 preliminary data, prior studies on preoperative information, and the clinical knowledge of five postoperative recovery nurses with at least five years of experience. Content validity was evaluated by an anesthesiology professor, a pediatric nursing professor, and three postoperative recovery nurses with at least 10 years of clinical experience, and the intervention was modified accordingly.

The intervention consisted of four sections: an introduction to build therapeutic rapport, a preoperative section, an operating room (OR) section, and a postoperative recovery section. Photographs of the actual preoperative, OR, and postoperative recovery environments were provided. The education was administered using a video featuring animated health care providers. In the introductory section, children were introduced to their nurses and informed that they would be given information on the process of their surgery. This was followed by a brief explanation of the surgery. Children were then asked whether they had fasted for surgery, were wearing their surgical gowns, had removed all accessories, and had visited the restroom. In the preoperative section, children were shown a photograph of the actual preoperative room and informed that their caregiver would be with them when they were taken to the OR. They were also made to perform a balloon breathing exercise to teach them how to take deep breaths. In the OR section, children were shown a photo of the OR, informed that they would be anesthetized during the surgery, and told how long the surgery would take. They were also shown a photo of the patient monitor used during the surgery and the mask used for induction of anesthesia, along with a brief explanation of why these devices are needed. In the postoperative recovery section, the children were informed that their caregivers would be with them in the recovery room. They were taught about postoperative discomfort and necessary precautions, such as using an oxygen mask and the balloon breathing technique to recover from anesthesia, and using an eye patch to protect the surgery site.

#### 2.3.2. Physiological Responses Indicating Anxiety

The children’s physiological responses were measured with the pulse oximeter unit of a patient monitor (Philips IntelliVue MP30, Hewlett-Packard Str. 2, Boeblingen, Germany). Their pulse was taken one hour before surgery and when the post-anesthesia recovery score was nine or higher after arrival in the postoperative recovery room.

#### 2.3.3. Behavioral Responses Indicating Anxiety

Children’s behavioral responses were measured using the Children’s Emotional Manifestation Scale (CEMS) [17]. This scale measures behavioral responses indicating anxiety across five subscales: facial expression, vocalization, activity, interaction, and level of cooperation. The children were observed before surgery and in the postoperative recovery room, and their displayed behaviors were scored on a five-point scale. The total scores range from 5 to 25; higher scores indicate higher anxiety levels. The Cronbach’s α was 0.92 at the time of development, 0.75 for preoperative anxiety behaviors, and 0.88 for postoperative anxiety behaviors.

### 2.4. Data Collection

#### 2.4.1. Identifying Anxiety Risk Factors and Determining Children’s Educational Needs

To identify risk factors and determine educational needs, we conducted interviews with six children who underwent strabismus surgery immediately before their discharge. They were asked whether they were experiencing pain rated three or higher on the Wong–Baker FACES Pain Rating Scale [18] and whether there were any side effects of general anesthesia, such as dizziness or vomiting. Children who were without these symptoms were chosen to participate in the interview. Data were collected during 23–30 May 2018. We asked open-ended questions and recorded the children’s responses ad verbum. Key questions were “What were you most afraid of when you were having surgery?” and “What were some things that made you uncomfortable or caused difficulties after your surgery?”

#### 2.4.2. Analysis of Educational Intervention Effectiveness

Data were collected from 19 June to 16 October 2018. The study was conducted in the same-day surgery ward and pediatric recovery room with cooperation from the corresponding departments. Data collection from the control and experimental groups was set for alternating days to prevent diffusion or contamination of experimental effects due to patients from both groups being admitted and discharged on the same day.

Children scheduled for surgery were selected based on their electronic medical records the day before surgery, and children admitted to the same-day surgery ward were enrolled. At baseline, general patient information was collected using emergency medical records and a questionnaire; additionally, by visiting the same-day surgery ward, preoperative anxiety was measured one hour before surgery. The educational intervention was administered by the researcher in the preoperative room. The control group was provided with the conventional verbal preoperative education, and the experimental group was shown the educational intervention video developed for the study on a tablet computer. To improve the effectiveness of the intervention, the researcher confirmed the children’s understanding after the intervention and held a question-and-answer session. The post-intervention survey consisted of scores in the following five categories: reflexes, breathing, circulation, consciousness, and skin color. Anxiety was indicated by a score of nine or higher upon arrival at the postoperative recovery room. To prevent researcher bias, a post-intervention survey using a checklist was conducted by the researcher and a research assistant (a nurse with at least five years of experience in the recovery room), who received training on the purpose of the study and CEMS, and interrater agreement was examined. To improve interrater reliability, observation training was conducted for five rounds, and 100% agreement was reached in round five. To minimize errors, the research assistant was blinded to participant allocation.

### 2.5. Data Analysis

SPSS Statistics software version 23 (IBM Corp., Armonk, NY, USA) was used to analyze data. The Shapiro–Wilk test was used to check the data for normality; as the data were not normally distributed, nonparametric tests were conducted. The data were analyzed as follows:

1. Participants’ general characteristics were analyzed with descriptive statistics, namely, real number and percentage and χ^2^ test.
2. Homogeneity between the experimental and control groups was tested using the Mann–Whitney U test.
3. The hypotheses were tested using the Mann–Whitney U test and the Wilcoxon signed-rank test to compare preoperative and postoperative anxiety between the groups. The differences in anxiety between them were analyzed using the Mann–Whitney U test.

### 2.6. Ethical Considerations

This study was conducted according to the guidelines of the Declaration of Helsinki and approved by the Pusan National University Yangsan Hospital Institutional Review Board. After obtaining an additional approval from the hospital review board (No. 05-2018-060, 5 May 2018), we conducted interviews with six children who underwent strabismus surgery. The effectiveness of the developed education program was analyzed after obtaining an additional approval (No. 05-2018-097, 19 June 2018). We guaranteed anonymity of data and informed participants of their right to withdraw from the study at any time. Both children and their parents provided informed consent.

## 3. Results

### 3.1. Risk Factors

The results of the interviews that surveyed the children’s anxiety risk factors and their educational needs were divided into “psychological anxiety”, “physical discomfort”, and “other.” Psychological anxiety was related to fear of the surgery itself, fear of the outcome of the surgery, fear of awakening during surgery, and uncertainty regarding the environment. The risk factors for physical discomfort included pain at the surgical site, using an eye patch or gauze, dizziness caused by general anesthesia, and sleep issues. Other risk factors included uncertainty regarding when and whether their parents would be present during the process (Table 1).

### 3.2. Homogeneity of General Characteristics

Fifteen participants each were assigned to the control and experimental groups, and the homogeneity of general characteristics is shown in Table 2.

There were seven boys (46.7%) and eight girls (53.3%) in the experimental group and five boys (33.3%) and 10 girls (66.7%) in the control group. Most children in the experimental group were aged nine or older (*n* = 6, 40.0%); in the control group, most were younger than six years (*n* = 7, 46.7%). There were nine cases of strabismus surgery (60.0%) and six cases of epiblepharon surgery for all participants. There were eight first-born children (53.3%), six second-born children (40.0%), and one third-born child (6.7%) in the experimental group and five first-born children (33.3%), nine second-born children (60.0%), and one third-born child (6.7%) in the control group. Six children in the experimental group (40.0%) and one in the control group (6.7%) had a history of surgery, while nine children in the experimental group (60.0%) and fourteen in the control group (93.3%) did not. Finally, eight children in the experimental group (53.3%) and five children in the control group (33.3%) had a history of hospitalization, while seven children in the experimental group (46.7%) and ten in the control group (66.7%) did not. There were no statistically significant differences in general characteristics between the two groups, and their homogeneity was confirmed.

### 3.3. Homogeneity of Baseline Study Parameters

The homogeneity of preoperative anxiety behaviors and pulse rate between the experimental and control groups was tested using the Mann–Whitney U test. Preoperative anxiety behaviors were homogeneous between the experimental group (median 10.00, inter quartile range (IQR) 2.00) and the control group (median 10.00, IQR 2.00) (Z = −0.63, *p* = 0.526). Preoperative pulse was also homogeneous between the experimental group (median 98.00, IQR 20.00) and the control group (median 99.00, IQR 18.00; Z = −0.21, *p* = 0.833) (Table 3).

### 3.4. Hypothesis Testing

Pulse rate changes in the experimental and control groups were analyzed using the Wilcoxon signed-rank test (Table 4). The experimental group showed a statistically significant change in pulse rate after the surgery compared to the baseline (Z = −2.45, *p* = 0.014); the control group also showed a statistically significant increase in pulse rate after the surgery compared to the baseline (Z = −2.68, *p* = 0.007). The differences in pulse rates between the two groups were analyzed using the Mann–Whitney U test. With a median of 8.00 in the experimental and control groups (Z = −0.04, *p* = 0.967), the groups did demonstrate a statistically significant difference in pulse rates.

The behavioral responses indicating anxiety in the experimental and control groups were analyzed using the Wilcoxon signed-rank test. The experimental group did not show a significant change in anxiety behaviors after the surgery compared to the baseline (Z = −1.50, *p* = 0.135). However, the control group showed a statistically significant increase in anxiety behaviors after the surgery compared to the baseline (Z = −3.33, *p* = 0.001). The differences in anxiety behaviors between the two groups were analyzed using the Mann–Whitney U test; statistically significant differences were found: a median of −1.00 in the experimental group and 6.00 in the control group (Z = −4.15, *p* < 0.001).

## 4. Discussion

We examined the effects of preoperative educational intervention on postoperative anxiety among children scheduled for same-day eye surgery. Both the experimental and control groups showed increased pulse rates after surgery, compared to the baseline, with no statistically significant differences between the two groups. This is similar to findings that pulse rates did not significantly differ in a study that provided a picture-based educational intervention [12] and a study that deployed a diversion intervention using a smartphone [15]. Furthermore, this result partially correlates with Yun, Kim, and Jung [16]: a preoperative program involving a nurse dressed as a clown was implemented, and the children’s physiological anxiety was measured after surgery, concluding that changes in systolic blood pressure were statistically significant, while changes in diastolic blood pressure and pulse rates were not. However, this contrasts with a study on children undergoing same-day surgery that found lower blood pressure and pulse rates in those who underwent a therapeutic preoperative program compared with those who received conventional information [19]. Yun, Kim, and Jung [16] also contrast with Hong and Jung [13] on children undergoing strabismus surgery; they found a statistically significant change in the pulse rate of the experimental group, which received animation-based information, compared to the control group. Anxiety triggers physiological responses to stress and threatening events, and findings indicate that anxiety activates the autonomic nervous system, involving sympathetic, parasympathetic, and endocrine stimulation, and provokes cardiovascular hypersensitivity, leading to increased heart rate, blood pressure, and respiratory rate, in the early phase [20]. On the basis of these findings, and because it can be easily measured without additional procedures, we used pulse rate as an indicator of physiological anxiety. However, it is difficult to compare the studies mentioned above with our study examining postoperative physiological anxiety as an outcome of preoperative intervention. Although increased pulse rate may be an outcome of surgery, inconsistencies among study findings hinder generalization and call for further replication studies. In addition, the variability of blood pressure based on the time of day must be considered. The pattern of normal blood pressure and pulse change during the day is as follows: lowest during sleep, rises with awakening in the morning, remains elevated during the day, and starts to decrease in the afternoon; the lowest point is between midnight and 3 a.m. In other words, blood pressure varies depending on the time of measurement [21].

Our study showed statistically significant differences in postoperative changes in anxiety behaviors between the experimental and control groups, confirming that preoperative educational intervention is effective in alleviating anxiety behaviors after surgery. This is consistent with previous findings that the experimental group showed greater reduction in anxiety behavior scores than did the control group after strabismus surgery [16]. It is also similar to findings that age-appropriate education reduces preoperative anxiety [22,23]. These results suggest that preoperative nursing interventions that involve the delivery of information are effective in reducing children’s anxiety and lowering their negative emotional behavioral responses through psychological preparation for surgery, which can help improve their coping skills.

In particular, we used a video-based educational intervention to reduce anxiety, and our results are consistent with a previous study that provided an audiovisual presentation to children undergoing same-day surgery [11]. Here, the group that received the experimental intervention showed the lowest preoperative anxiety score, while the control group, which did not receive the preoperative intervention, showed the highest maladaptive behavior score, seven days after discharge. Moreover, regarding findings that gradual visual exposure to an unfamiliar environment effectively reduces anxiety [24] and that the delivery of information using pictures reduces anxiety in children [12], we can infer that visual aids are more effective for relieving anxiety than verbal delivery alone. This suggests that, in addition to relieving preoperative and postoperative anxiety, preoperative intervention can facilitate children’s return to normalcy and reduce regressive behavioral changes triggered by stress.

Our preoperative educational intervention was based on the clinical experiences of health care providers, objective nursing knowledge, and the experiences of children undergoing same-day eye surgery. In our survey of the experiences of children undergoing same-day eye surgery, the most common risk factor for anxiety was fear of the surgery itself, as well as its outcomes. In particular, children were worried that they might lose their vision. As such, we included a brief explanation regarding the surgery they would be undergoing, clarifying that they could lose their vision after surgery but that the condition would be temporary; this seems to have helped reduce the children’s anxiety. Furthermore, the children were anxious regarding waking up during anesthesia. To reduce this anxiety, we included information on the patient monitoring devices used during the anesthesia process. In addition, based on the findings that gradual visual exposure to an unfamiliar environment effectively reduces anxiety [24] and that the delivery of information using pictures reduces anxiety in children [12], we showed the participants photos of the actual surgical areas to familiarize them with the environment. This seems to have contributed to alleviating their anxiety. These results demonstrate that visual aids are more effective than the conventional verbal delivery of information for reducing anxiety in children undergoing surgery. Risk factors for discomfort included pain at the surgical site, discomfort from the eye patch or gauze, dizziness from anesthesia, and sleep issues. Therefore, teaching participants deep breathing techniques to facilitate recovery from anesthesia and teaching them why they need to protect their eyes with an eye patch or gauze seems to have helped reduce anxiety [4]. Participants were also curious about when their parents would be with them during the surgery process, so we provided that specific information. Thus, the participants were able to predict when they would be with their parents. These results support findings that the presence of caregivers in the recovery room effectively relieves anxiety in children and adolescents [25] and that the presence of parents may provide physiological and physical stability to children undergoing surgery [5]. Hence, identifying the factors pertaining to anxiety and educational needs in children who have undergone surgery and reflecting them in educational interventions before surgery seems to have contributed to reducing children’s anxiety behaviors.

For future research, we suggest the following. It will be necessary to develop interventions to reduce parents’ anxiety or to teach them strategies to help their children in both the pre-intervention and post-intervention phase. Moreover, a longitudinal approach could be useful for comprehensively understanding anxiety in children who undergo surgery, perhaps by observing them until the next check-up or following up on them post-intervention. In the future, there is a need for research exploring coping strategies that reduce children’s anxiety levels, which could help increase intervention efficacy.

## 5. Conclusions

This study is significant as it developed and assessed the effectiveness of an informational intervention based on children’s anxiety risk factors and their educational needs. This study also confirmed that preoperative educational intervention is effective in postoperative anxiety relief; hence, implementing the intervention in clinical practice will contribute to promoting evidence-based nursing.

This study is limited by its small sample size and the fact that it only included pediatric patients from a single tertiary hospital. As this may affect the results of hypothesis testing, subsequent studies should utilize a larger sample to obtain more accurate results. In addition, most same-day surgeries are either minor or are minimally invasive; children’s displayed anxiety and subsequent coping strategies may differ with more invasive surgeries. Thus, we recommend that subsequent studies utilize samples including children undergoing various types of surgeries.

## Figures and Tables

**Table 1:** Interview of educational needs.

Category	Subcategory	Content
Psychological anxiety	Surgery procedure and prognosis	I’m very scared of having surgery.
		I am afraid that I will not be able to see my eyes after surgery.
		I am afraid that the results of the surgery will not be good.
	Awakening during anesthesia	I’m worried that getting anesthesia means going to sleep forever.
		I was scared of waking up during the surgery.
	New environment	I am nervous because of the surrounding environment.
Physical discomfort	Surgical site	The surgery area is sore.
		My eyes are itchy.
	Treatment	The eye patch is too tight.
		I am uncomfortable because of the gauze.
	Side effects of general anesthesia	I am dizzy.
		I was falling asleep, but the medical staff said that I shouldn’t sleep.
Other	Presence of parents	Can I stay with my mom?

**Table 2:** Homogeneity test for participants’ general characteristics.

Characteristic	Category	Experimental Group (*n* = 15) *n* (%)	Control Group (*n* = 15) *n* (%)	χ²	*p*
Gender	Boy	7 (46.7)	5 (33.3)	0.56	0.456
	Girl	8 (53.3)	10 (66.7)		
Age (years)	<6	4 (26.7)	7 (46.7)	1.33	0.653
	≥6, <9	5 (33.3)	4 (26.7)		
	≥9	6 (40.0)	4 (26.7)		
Type of surgery	Strabismus	9 (60.0)	9 (60.0)	0.00	1.000
	Epiblepharon	6 (40.0)	6 (40.0)		
Birth order	First	8 (53.3)	5 (33.3)	1.29	0.715
	Second	6 (40.0)	9 (60.0)		
	Third	1 (6.7)	1 (6.7)		
Surgical experience	Yes	6 (40.0)	1 (6.7)	4.66	0.080
	No	9 (60.0)	14 (93.3)		
Hospitalization experience	Yes	8 (53.3)	5 (33.3)	1.22	0.269
	No	7 (46.7)	10 (66.7)		

**Table 3:** Homogeneity of baseline study variable.

Variable	Experimental Group (*n* = 15)		Control Group (*n* = 15)		Z	*p*
	Median	IQR	Median	IQR		
Behavioral responses to anxiety	10.00	2.00	10.00	2.00	–0.63	0.526
Pulse	98.00	20.00	99.00	18.00	–0.21	0.833

IQR = interquartile range.

**Table 4:** Homogeneity of baseline study variables.

	Variable	Pre-Surgery		Post-Surgery		Z ^†^	*p*	Pre–Post Difference		Z ^‡^	*p*
		Median	IQR	Median	IQR			Median	IQR		
Pulse rate	Experimental Group	98.00	20.00	112.00	22.00	–2.45	0.014	8.00	10.00	−0.04	0.967
	Control Group	99.00	18.00	112.00	20.00	–2.68	0.007	8.00	10.00		
Behavioral responses to anxiety	Experimental Group	10.00	2.00	9.00	2.00	–1.50	0.135	−1.00	2.00	−4.15	<0.001
	Control Group	10.00	2.00	16.00	4.00	–3.33	0.001	6.00	3.00		

IQR = Interquartile range. ^†^: Wilcoxon signed-rank test. ^‡^: Mann–Whitney U test.

## Data Availability

The data are not publicly available owing to privacy or ethical restrictions. The data that support the findings of this study are available from the corresponding author upon reasonable request.
